# Prevalence of pulmonary embolism in patients with COVID-19 pneumonia and high D-dimer values: A prospective study

**DOI:** 10.1371/journal.pone.0238216

**Published:** 2020-08-25

**Authors:** Alberto Alonso-Fernández, Nuria Toledo-Pons, Borja G. Cosío, Aina Millán, Néstor Calvo, Luisa Ramón, Sara Hermoso de Mendoza, Daniel Morell-García, Josep Miquel Bauça-Rossello, Belén Núñez, Jaume Pons, Juan A. Palmer, Luisa Martín, María Peñaranda, Joan A. Pou, Jaume Sauleda, Ernest Sala-Llinas

**Affiliations:** 1 Department of Pneumology, University Hospital Son Espases, Palma de Mallorca, Spain; 2 CIBER Enfermedades Respiratorias, Madrid, Spain; 3 Institut d’Investigació Sanitària Illes Balears (IdISBa), University Hospital Son Espases, Palma de Mallorca, Spain; 4 Radiodiagnostic Department, University Hospital Son Espases, Palma de Mallorca, Spain; 5 Department of Laboratory Medicine, University Hospital Son Espases, Palma de Mallorca, Spain; 6 Cardiology Department, University Hospital Son Espases, Palma de Mallorca, Spain; 7 Department of Internal Medicine, University Hospital Son Espases, Palma de Mallorca, Spain; BronxCare Health System, Affiliated with Icahn School of Medicine at Mount Sinai, NY, USA, UNITED STATES

## Abstract

**Introduction:**

Coronavirus disease 2019 (COVID-19) pneumonia is associated to systemic hyper-inflammation and abnormal coagulation profile. D-dimer elevation is particularly frequent, and values higher than 1μg/mL have been associated with disease severity and in-hospital mortality. Previous retrospective studies found a high pulmonary embolism (PE) prevalence, however, it should be highlighted that diagnoses were only completed when PE was clinically suspected.

**Material and methods:**

Single-center prospective cohort study. Between April 6^th^ and April 17^th^ 2020, consecutive confirmed cases of COVID-19 pneumonia with D-dimer >1 μg/mL underwent computed tomography pulmonary angiography (CTPA) to investigate the presence and magnitude of PE. Demographic and laboratory data, comorbidities, CTPA scores, administered treatments, and, clinical outcomes were analysed and compared between patients with and without PE.

**Results:**

Thirty consecutive patients (11 women) were included. PE was diagnosed in 15 patients (50%). In patients with PE, emboli were located mainly in segmental arteries (86%) and bilaterally (60%). Patients with PE were significantly older (median age 67.0 (IQR 63.0–73.0) vs. 57.0 (IQR 48.0–69.0) years, p = .048) and did not differ in sex or risk factors for thromboembolic disease from the non-PE group. D-dimer, platelet count, and, C reactive protein values were significantly higher among PE patients. D-dimer values correlated with the radiologic magnitude of PE (p<0.001).

**Conclusions:**

Patients with COVID-19 pneumonia and D-dimer values higher than 1 μg/mL presented a high prevalence of PE, regardless of clinical suspicion. We consider that these findings could contribute to improve the prognosis of patients with COVID-19 pneumonia, by initiating anticoagulant therapy when a PE is found.

## Introduction

The World Health Organization (WHO) has declared coronavirus disease 2019 (COVID-19) as a pandemic and a major public health emergency [1]. The clinical spectrum of the disease caused by the novel severe acute respiratory syndrome coronavirus 2 (SARS-CoV-2) is wide-ranging, from asymptomatic infection to acute respiratory distress syndrome (ARDS) with high mortality [2]. Risk factors for severe disease and death have been reported in retrospective cohorts [3–6]. Among them, older age, a higher sequential organ failure assessment (SOFA) score, and a D-dimer higher than 1 μg/mL at admission were risk factors for death [3]. Patients with severe COVID-19 may also exhibit features of systemic hyper-inflammation or cytokine storm [7]. Besides, coagulation disorders are frequently encountered among COVID-19 patients, especially among those with severe disease [3,5]. This has been further confirmed in larger studies showing that nearly 50% of patients with laboratory confirmed COVID-19 infection had elevated D-dimer and fibrin degradation products, being the elevation more pronounced among severe cases [2,8]. In addition, in a multicenter retrospective cohort study from China, increased D-dimer levels (>1μg/mL) were significantly associated with in-hospital death in the multivariable analysis (p = 0.003) [3]. On the other hand, it is worth noting that in a retrospective study, including 449 severe COVID-19 patients in Wuhan, low molecular weight heparin administration among patients with markedly elevated D-dimer was significantly associated with better 28-day overall survival [9].

First series of autopsies in COVID-19 patients showed thrombosis in small pulmonary vessels [10], and first clinical cases with PE in COVID-19 patients has already been published [11]. Interestingly, Klok et al. [12] found, in a retrospective study in 184 intensive care unit (ICU) patients with severe COVID-19 pneumonia, a high prevalence of thrombotic complications and, by far, pulmonary embolism (PE) was the most frequent. Similarly, some other retrospective studies found a high frequency of PE in COVID-19 patients [12–16]. However, the real prevalence was not known, since computed tomography pulmonary angiography (CTPA) was only performed when thrombotic complications were clinically suspected.

Overall, all the above seems to indicate that D-dimer elevation may be common in patients with severe form of COVID-19 infection. It is reasonable to conceive that COVID-19 infected patients could be at high risk for venous thromboembolic events. Although the published data are very limited, it seems rational to suggest that D-dimer evaluation could offer useful information for the search of PE in severe COVID-19 infected patients. Accordingly, we aimed to evaluate prospectively the prevalence of PE in patients admitted to hospital with COVID-19 pneumonia and D-dimer >1μg/mL. As a secondary objective we evaluated clinical, radiological and biological variables that potentially could be related with this event.

## Material and methods

### Study design and participants

We performed a single-center prospective cohort study (Hospital Universitario Son Espases, Palma de Mallorca, Spain) from April 6 to April 17, 2020. We selected consecutive adult patients with confirmed COVID-19 pneumonia admitted to the hospital and with at least one D-dimer value higher than 1 μg/mL during hospitalization. The diagnosis of COVID-19 pneumonia was done according to the case definition established by WHO interim guidance.^17^ Patients were excluded if they fulfilled at least one of the following exclusion criteria: (1) unwillingness or inability to participate in the study; (2) previous allergic reactions to iodinated contrast media; (3) previous anticoagulant treatment in the three months prior to admission; (4) CTPA performed before D-dimer rising above 1 μg/mL and/or (5) any other concurrent severe medical condition that would, in the investigator’s judgment, contraindicate patient participation in the study. The clinical suspicion of PE was not and exclusion criterion.

All patients were followed up until hospital discharge, death or until April 17th 2020, whichever came first. The STROBE standards for reporting observational studies were followed.

### Description of investigations undertaken

Epidemiological, demographic, clinical and laboratory examinations were collected in all subjects by the time of admission. The recorded data included time from symptoms onset to hospital admission, time from symptoms onset to CPTA, medical treatment during hospitalization, VTE prophylaxis, respiratory support, clinical outcomes (acute respiratory failure, arrhythmia, intensive care unit admission or death) and permanent and temporary PE risk factors. Temporary PE risk factors were considered if they were present within the 3 months before the admission.

Laboratory data included complete blood count (Cell-Dyn Sapphire platform, Abbott Diagnostics), coagulation (including D-dimer), kidney and, liver function tests, collected at admission, on days 1, 3 and then at the discretion of the attending physician onwards. In addition, we analysed in each patient, baseline, peak and prior to CTPA values of the following biomarkers: D-dimer (ACL TOP 700, Instrumentation Laboratory), C-reactive protein (CRP), lactate dehydrogenase (LDH), erythrocyte sedimentation rate (ESR), ferritin, lymphocytes and, neutrophil-to-lymphocyte ratio (NLR). The D-dimer-to-ferritin, D-dimer-to-LDH and D-dimer-to-CRP ratios were measured at baseline moment. Besides, we measured high sensitive troponin I, interleukin-6 (IL-6, by ELISA, R&D systems), N-terminal pro hormone B-type natriuretic peptide (NT pro-BNP) (Test 1 THL Module, ALI FAX; Architect platform, Abbott Diagnostics) and, fibrinogen values.

The severity of pneumonia was defined according to the CURB-65 score [17].

Laboratory confirmation of SARS-CoV-2 infection was defined as a positive result of real-time reverse transcriptase–polymerase chain reaction (RT-PCR) assay of nasal and pharyngeal swabs [18].

### Computed tomography pulmonary angiography

The CTPA was requested per protocol only if the D-dimer determination was higher than 1ug/mL, regardless of symptoms. Diagnosis of PE was performed by an expert radiologist based on direct visualization of the endoluminal thrombus in the pulmonary arteries. In order to provide a quantitative assessment of the magnitude of the embolism, the pulmonary artery obstruction index (PAOI) was calculated according to the formula proposed by Qanadli et al. [19].

### Ethics statement

The Institutional Ethic Committee of the Balearic Islands approved the study (IB 4197/20 PI), and all subjects gave their written informed consent. Only, those patients with a severe and critical clinical condition gave verbal consent with at least two witnessed.

### Statistical analysis

Descriptive statistics included frequency analysis (percentages) for categorical variables and medians and interquartile ranges (IQRs) for continuous variables. Comparisons were determined by Mann-Whitney U or Kruskall-Wallis test for continuous variables as appropriate and by the use of the χ2 test or Fisher exact test for categorical variables.

Rho Spearman correlation was used in order to assess significant relationship between severity PE markers (PAOI) and inflammatory biomarkers. Receiver‐operating characteristic (ROC) curve analysis was performed to identify thresholds, and the sensitivity and specificity of different cut-off points of the inflammatory markers for the discrimination of PE. Differences are considered statistically significant at 2-tailed p<0.05. The statistical software used to analyse the data was SPSS v.26 (IBM).

## Results

### COVID-19 pneumonia population

**Fig 1:**
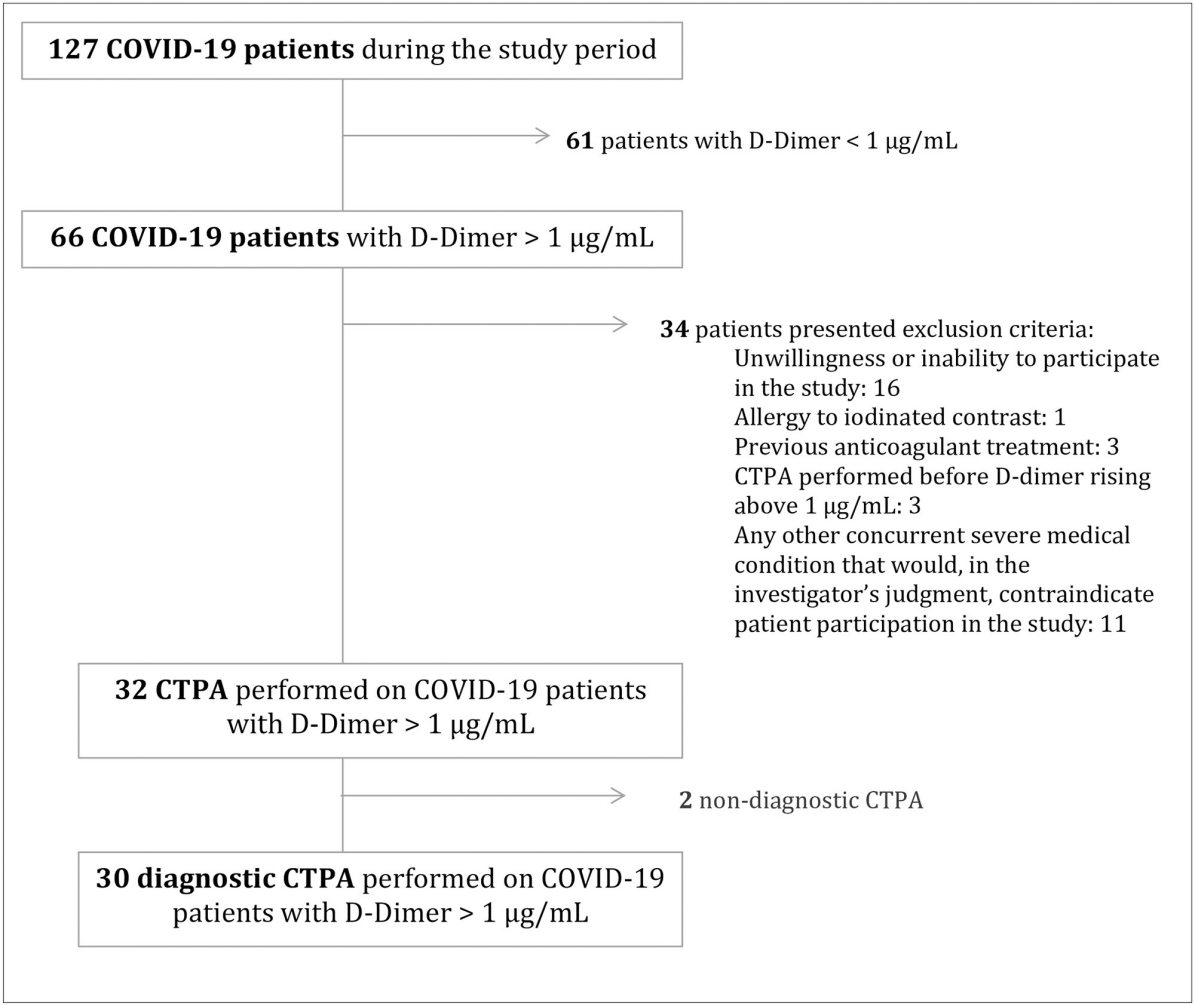
Flow-chart. Abbreviations: CTPA, Computed tomography pulmonary angiography; PE, pulmonary embolism.

One hundred and twenty-four COVID-19 confirmed patients were hospitalized during the study period (6 to 17 April). Sixty-three of those patients (50.8%) presented a D-dimer > 1 μg/mL at some time-point during the admission. Thirty-one patients presented at least one exclusion criteria. The CTPA was performed on 32 patients, but 2 of them were excluded for technical reasons (Fig 1).

Finally, 30 patients were included in the analysis. The anthropometric and clinical characteristics are described in Table 1. The median age was 64.5 (IQR 55.8–71.3) years and 63.3% were males. The median time from symptoms onset to hospital admission was 8 (IQR 4.8–14.0) days. At the time of admission the median fractional inspired oxygen (FiO2)/partial pressure of arterial blood oxygen (PaO2) ratio was 264 (IQR 211–343) and the median CURB-65 score was 1 (IQR 1–2). Eleven patients (37.9%) were admitted in the intensive care unit (ICU) at certain time-point of their clinical evolution.

**Table 1:** Anthropometric and clinical characteristics of patients admitted because of COVID-19 pneumonia at baseline (all patients) and according to the outcome (with and without Pulmonary embolism).

	All patients (n = 30)	PE patients (n = 15)	Non-PE patients (n = 15)	p value
**Age, yr**.	64.5 (55.8–71.3)	67.0 (63.0–73.0)	57.0 (48.0–69.0)	0.048
**Gender, men, n (%)**	19 (63.3%)	10 (66.7%)	9 (60.0%)	0.704
**Body mass index, Kg/m2**	28.2 (26.4–31.1)	28.9 (26.6–31.2)	27.5 (25.9–31.5)	0.727
**Cardiovascular disease, n (%)**	12 (40.0%)	8 (53.3%)	4 (26.7%)	0.136
**Arrhythmia, n (%)**	0 (0.0%)	0 (0.0%)	0 (0.0%)	NA
**Respiratory chronic disease, n (%)**	5 (16.7%)	4 (26.7%)	1 (6.7%)	0.329
**Previous antiplatelet treatment, n (%)**	7 (23.3%)	3 (20.0%)	4 (26.7%)	1.000
**Time from illness onset to hospital admission, days**	8.0 (4.8–14.0)	11.0 (7.0–15.0)	7.0 (4.0–10.0)	0.080
**Time from illness onset to CTPA, days**	20.0 (14.8–24.3)	19.0 (15.0–22.0)	21.0 (14.0–25.0)	0.802
**Current or former smokers, n (%)**	7 (23.3%)	3 (20.0%)	4 (26.7%)	1.000
**Smoking, Pack-year**	30.0 (28.5–40.0)	30.0 (27.0–0.0)	40.0 (40.0–40.0)	0.067
**Symptoms**				
Cough, n (%)	27 (90.0%)	14 (93.3%)	13 (86.7%)	1.000
Fever, n (%)	26 (86.7%)	14 (93.3%)	12 (80.0%)	0.597
Dyspnea, n (%)	19 (63.3%)	10 (66.7%)	9 (60.0%)	0.704
Hemoptysis, n (%)	0 (0.0%)	0 (0.0%)	0 (0.0%)	NA
Chest pain, n (%)	2 (6.7%)	1 (6.7%)	1 (6.7%)	1.000
Physical examination*				
Respiratory rate, breaths per min	22.0 (18.0–25.5)	24.0 (18.0–26.0)	22.0 (17.8–26.0)	0.775
Heart rate, beats per min	90.0 (77.3–109.3)	90.0 (75.0–109.0)	92.0 (84.0–110.0)	0.319
Systolic BP, mm Hg	130.0 (117.0–139.8)	130.0 (117.0–142.0)	132.0 (121.0–139.0)	0.787
Diastolic BP, mm Hg	72.5 (63.8–85.0)	75.0 (64.0–89.0)	71.0 (59.0–81.0)	0.299
Temperature, °C	37.1 (36.1–37.7)	37.1 (36.1–37.7)	37.1 (35.7–37.8)	0.851
Lower limb edema, n (%)	3 (10.0%)	2 (13.3%)	1 (6.7%)	1.000
**CURB 65**	1.0 (1.0–2.0)	1.0 (1.0–2.0)	1.0 (0.0–2.0)	0.572
**PE risk factors**				
Diabetes mellitus, n (%)	8 (26.7%)	4 (26.7%)	4 (26.7%)	1.000
Cardiovascular disease, n (%)	12 (40.0%)	8 (53.3%)	4 (26.7%)	0.136
Chronic respiratory failure, n (%)	1 (3.3%)	1 (6.7%)	0 (0.0%)	1.000
Varicose veins, n (%)	1 (3.3%)	1 (6.7%)	0 (0.0%)	1.000
Chronic venous insufficiency, n (%)	1 (3.3%)	0 (0.0%)	1 (6.7%)	1.000
Neoplasm, n (%)	1 (3.3%)	1 (6.7%)	0 (0.0%)	1.000
Previous VTE, n (%)	1 (3.3%)	1 (6.7%)	0 (0.0%)	1.000
Chronic heart failure, n (%)	1 (3.3%)	1 (6.7%)	0 (0.0%)	1.000
Ischemic heart disease, n (%)	3 (10.0%)	1 (6.7%)	2 (13.3%)	1.000
Pregnancy or puerperium, n (%)	1 (3.3%)	0 (0.0%)	1 (6.7%)	1.000
Obesity, n (%)	9 (31.0%)	4 (26.7%)	5 (35.7%)	0.699
**One or more known risk factors for PE, n (%)**	16 (53.3%)	9 (60.0%)	7 (46.7%)	0.464
**Treatment in hospital**				
**Oxygen therapy**				
Maximum FiO2	0.7 (0.3–1.0)	0.4 (0.4–1.0)	1.0 (0.3–1.0)	0.546
HFNC, n (%)	4 (13.3%)	1 (6.7%)	3 (20.0%)	0.597
NIV, n (%)	0 (0.0%)	0 (0.0%)	0 (0.0%)	NA
IMV, n (%)	7 (23.3%)	4 (26.7%)	3 (20.0%)	1.000
Azithromycin, n (%)	11 (73.3%)	3 (60.0%)	8 (80.0%)	0.560
Hydroxychloroquine, n (%)	27 (90.0%)	14 (93.3%)	13 (86.7%)	1.000
Lopinavir + Ritonavir, n (%)	17 (85.0%)	8 (80.0%)	9 (90.0%)	1.000
Tocilizumab, n (%)	11 (36.7%)	4 (26.7%)	7 (46.7%)	0.255
Other biological therapy, n (%)	1 (3.3%)	1 (6.7%)	0 (0.0%)	1.000
Systemic corticosteroids, n (%)	17 (56.7%)	9 (60.0%)	8 (53.3%)	0.712
**Clinical outcomes**				
Acute respiratory failure, n (%)	15 (50.0%)	9 (60.0%)	6 (40.0%)	0.273
Arrhythmia, n (%)	2 (6.7%)	2 (13.3%)	0 (0.0%)	0.482
ICU admission, n (%)	11 (37.9%)	5 (33.3%)	6 (42.9%)	0.597
Death, n (%)	0 (0.0%)	0 (0.0%)	0 (0.0%)	NA

Values represent percentage or median (IQR) according to its distribution. Abbreviations: CPTA, computed tomography pulmonary angiography; BP, blood pressure; PE, pulmonary embolism; VTE, Venous thromboembolism; IVF, in vitro fertilization; FiO2, fractional inspired oxygen; HFNC, High Flow Nasal Cannula; NIV, non-invasive ventilation; IMV, invasive mechanical ventilation; ICU, intensive care unit.

### PE prevalence among patients with COVID-19 pneumonia

The prevalence of PE was 50% (15 patients). One patient presented one strong risk factor for PE (previous medical history of deep vein thrombosis (DVT)). But, even after excluding this patient from the analysis, the prevalence of PE in the remaining population was 48.3%, being in this case, a population without any strong risk factor for PE.

### PE and non-PE patients’ comparison

PE patients were significantly older than patients without evidence of PE (median age (IQR) 67.0 (63.0–73.0) vs. 57.0 (48.0–69.0) years, respectively, p = 0.048). The remaining anthropometric and clinical characteristics, physical examination, PE risk factors, and treatment during hospitalization were not significantly different between cases with PE and subjects without PE (Table 1). Nine patients with at least one minor VTE risk factor were found to have PE, and seven patients with at least one minor risk factor did not show evidence of PE (p>0.05). Only one patient presented one major risk factor (5%, p>0.05) as mentioned above.

The proportion of patients with PE diagnosed during the first days (when laboratory data was obtained by protocol) was higher (66.7%) than the proportion of PE patients diagnosed during follow-up (when laboratory data was obtained at the discretion of the clinician) (42.9%).

All PE patients started anticoagulation after being diagnosed of PE. Fourteen out of 15 non-PE patients and 12 PE patients received thromboprophylaxis with enoxaparin (40 mg per day) by the time of admission. The remaining 3 PE subjects were diagnosed of PE at the time of admission, and they were started on anticoagulant therapy from the first day.

### Radiological findings

The median and IQR of the COVID-19 pneumonia CT severity score was 10 (5.8–13). This score did not present differences between the two subpopulations (PE 10 (IQR 5.0–15.0); non-PE 10 (IQR 7.0–13.0); p = 0.75). Regarding the PE radiological characteristics, the vascular allocation of emboli showed a predominantly peripheral and bilateral (60%) distribution, affecting mainly segmental and subsegmental arteries (53% and 7%, respectively). The overall PAOI was 15% (IQR 8–25%).

### Laboratory findings

The baseline laboratory findings and inflammatory and PE biomarkers are shown in Table 2 and S1 Table. Patients with COVID-19 pneumonia showed lymphopenia (1.2 (IQR 0.8–1.4) lymphocytes 10^3^/μL) at the time of admission.

**Table 2:** Initial inflammatory profile and pulmonary embolism biomarkers of patients admitted because of COVID-29 pneumonia with and without Pulmonary embolism.

	All patients (n = 30)	PE patients (n = 15)	Non-PE patients (n = 15)	p value
**LDH**				
Baseline, U/L	356.5 (280.3–479.8)	376.0 (281.0–477.0)	323.0 (268.0–611.0)	0.724
Peak, U/L	434.0 (317.5–609.0)	434.0 (319.0–590.0)	434.0 (268.0–611.0)	0.836
Prior to CTPA, U/L	341.0 (267.5–408.8)	331.0 (281.0–393.0)	361.0 (229.0–432.0)	0.868
**CRP**				
Baseline, mg/dL	13.4 (6.0–25.4)	10.7 (7.2–20.3)	19.2 (5.5–26.8)	0.604
Peak, mg/dL	18.7 (8.3–25.5)	10.7 (7.6–21.7)	19.2 (10.1–27.8)	0.272
Prior to CTPA, mg/dL	2.3 (0.7–7.5)	4.7 (1.3–9.2)	0.9 (0.2–4.5)	0.036
**ESR**				
Baseline, mm/h	67.0 (52.8–103.3)	70.0 (55.8–101.3)	64.0 (44.5–103.3)	0.625
Peak, mm/h	75.5 (59.8–107.5)	74.0 (62.0–106.8)	77.5 (55.3–109.8)	0.979
Prior to CTPA, mm/h	59.0 (44.0–77.3)	64.0 (55.8–91.5)	55.5 (30.0–69.5)	0.173
**D-dimer**				
Baseline, μg/mL	0.6 (0.3–3.6)	2.7 (0.4–10.0)	0.3 (0.2–1.0)	0.010
Peak, μg/mL	3.2 (2.0–9.5)	3.4 (2.6–11.2)	2.3 (1.6–7.3)	0.065
Prior to CTPA, μg/mL	2.4 (1.3–4.4)	2.6 (1.8–7.1)	1.6 (0.6–3.5)	0.071
**Ferritin**				
Baseline, ng/mL	846.0 (252.3–1614.0)	658.0 (256.0–994.0)	1477.0 (217.0–2290.0)	0.290
Peak, ng/mL	1375.0 (413.8–2618.8)	716.0 (404.0–2432.0)	1866.0 (545.0–3299.0)	0.130
Prior to CTPA, ng/mL	668.0 (277.5–1184.5)	658.0 (267.0–932.0)	764.0 (281.0–2409.0)	0.330
**Platelets**				
Baseline, 10^3^/μL	216.0 (171.3–315.5)	298.0 (181.0–338.0)	184.0 (165.0–256.0)	0.034
Peak, 10^3^/μL	381.0 (297.8–492.5)	350.0 (297.0–489.0)	405.0 (319.0–519.0)	0.372
Prior to CTPA, 10^3^/μL	288.0 (254.8–381.0)	292.0 (272.0–310.0)	280.0 (245.0–390.0)	1.000
**Lymphocytes**				
Baseline, 10^3^/μL	0.96 (0.6–1.27)	1.05 (0.59–1.29)	0.95 (0.6–1.23)	0.820
Peak*, 10^3^/μL	0.5 (0.4–0.9)	0.5 (0.4–1.0)	0.6 (0.4–0.8)	0.983
Prior to CTPA, 10^3^/μL	1.4 (0.8–1.7)	1.0 (0.7–1.5)	1.6 (1.0–2.0)	0.093
**NLR**				
Baseline	7.1 (3.8–10.8)	7.6 (4.5–10.8)	5.3 (3.8–11.9)	0.494
Peak	13.6 (5.7–22.8)	15.9 (9.9–26.7)	12.2 (4.6–22.7)	0.604
Prior to CTPA	4.6 (1.8–8.0)	5.0 (2.6–10.9)	2.8 (1.6–7.7)	0.101
**IL-6**, pg/mL	59.0 (21.6–156.0)	36.0 (20.7–89.4)	59.5 (41.2–225.6)	0.409
**NT-pro BNP**, pg/mL	190.5 (97.5–3353)	273.0 (139.0–851.0)	108.0 (89.0–284.0)	0.106
**hs Troponin I**, ng/L	6.0 (3.6–16.2)	9.2 (5.0–18.7)	4.2 (3.0–15.4)	0.135
**Fibrinogen**, mg/dL	592.0 (399.8–785.5)	616.0 (390.0–824.0)	523.0 (401.0–733.0)	0.494

Values represent median (IQR). Baseline, first variable value; Peak, maximum value; Peak*, minimum value; Previous to CTPA, previous value to CTPA. Abbreviations: CTPA, computed tomography pulmonary angiography; LDH, lactate dehydrogenase; CRP, C-reactive protein; ESR, erythrocyte sedimentation rate; IL-6, interleukin-6; NT-proBNP, N-terminal pro hormone B-type natriuretic peptide; NLR, neutrophil-to-lymphocyte ratio.

Higher baseline platelet count (298 (IQR 181–338) vs 184 (IQR 165–256) 10^3^/μL), higher D-Dimer baseline values (2.7 (0.4–10.0) vs 0.3 (0.2–1.0) μg/mL) and higher CRP values prior to CTPA (4.7 (1.3–9.2) vs 0.9 (0.2–4.5) mg/dL) were found in PE patients compared with non-PE patients (p value for all comparisons<0.05). On the other hand, it is worth noting that, despite the results were not statistically significant, there were also differences in the D-Dimer peak values and D-dimer values prior to CTPA, being this biomarker higher in the PE group (3.4 (IQR 2.6–11.2) vs 2.3 (IQR 1.6–7.3) μg/mL, p = 0.07 and 2.6 (IQR 1.8–7.1) vs 1.6 (0.6–3.5) μg/mL, p = 0.07, respectively). Moreover, the baseline D-dimer-to-ferritin, D-dimer-to-LDH and D-dimer-to-CRP ratios were significantly higher in PE patients compared with non-PE subjects (S1 Fig).

The ROC analysis carried out on the different peak inflammatory and PE biomarkers revealed that the highest area under the curve (AUC) was reached by peak D-dimer, with an AUC of 0.68 (95% CI 0.51–0.89; p = 0.065). Different cut-off points of peak D-dimer were tested in order to analyze the sensitivity and specificity reached in each one. D-dimer value >2.5 μg/mL was predictive of PE with a sensitivity of 80% and a specificity of 51%. S2 Table summarizes sensitivity, specificity, and positive and negative predictive values of different cut-off points of peak D-dimer (>1, >1.5, >2, and, >2.5 μg/mL).

**Fig 2:**
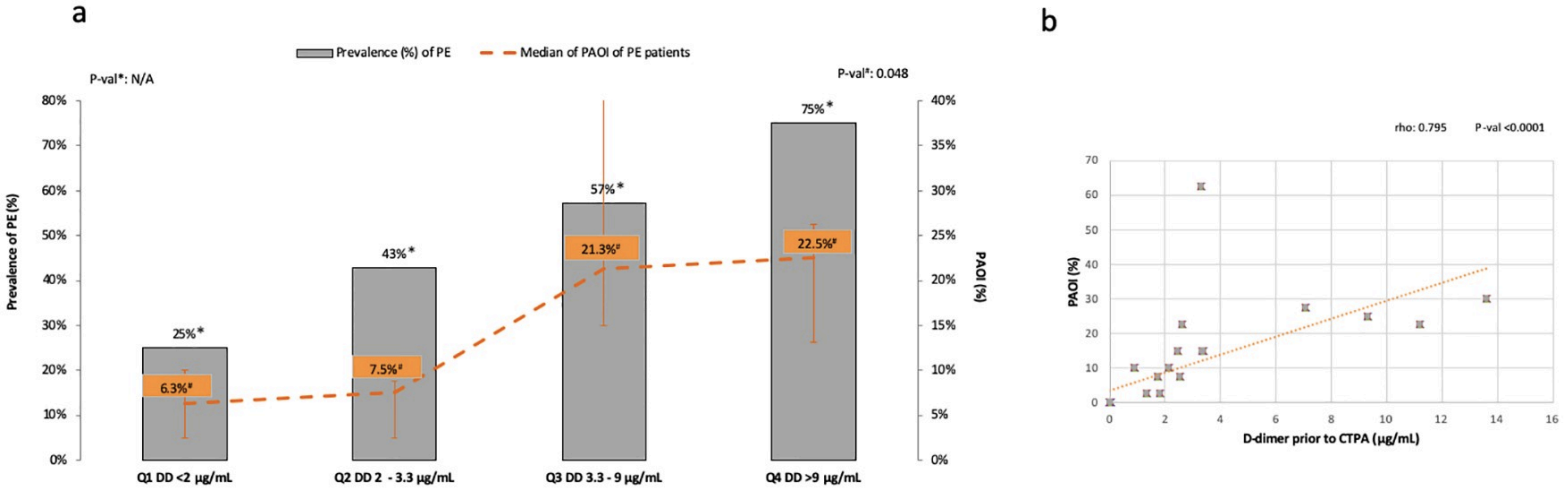
Relation between PAOI and D-dimer. **a**. PE prevalence and PAOI median of PE patients divided by D-dimer quartiles of the entire population. **b**. D-Dimer and PAOI correlation in PE patients. Abbreviations: a. Prev., prevalence; PAOI, pulmonary arterial obstruction index; PE, pulmonary embolism; Q1, first quartile; Q2, second quartile; Q3, third quartile; Q4, fourth quartile; DD, D-dimer; NA, not applicable. b. PAOI, pulmonary arterial obstruction index; CTPA, computed tomography pulmonary arteriography.

**Fig 3:**
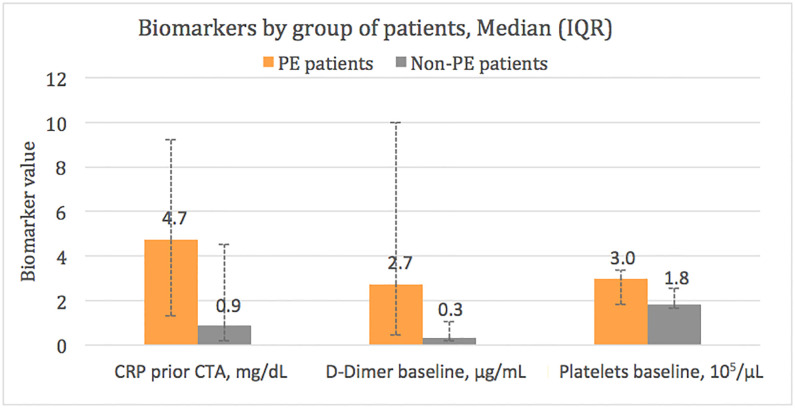
Significant biomarker differences between PE and non-PE populations. Abbreviations: PE, pulmonary embolism; IQR, interquartile range; CTPA, computed tomography pulmonary angiography.

Bivariate analysis showed significant correlations between the PAOI and peak (rho = 0.592, p = 0.020), and prior to CTPA (rho = 0.795, p<0.0001) D-dimer values, CRP prior to CTPA levels (rho = 0.518, p = 0.048), and baseline (rho = 0.574, p = 0.025), and, peak lymphocytes count (rho = 0.605, p = 0.017) (Figs 2 and 3).

## Discussion

In this study, we have shown that patients with COVID-19 pneumonia and D-dimer levels higher than 1 μg/mL had a remarkably high prevalence of PE. In addition, we found that patients with PE were older and had higher inflammatory (platelet counts, CRP) and procoagulant (D-dimer) markers that correlated with the extend of the thromboembolic episode compared to those patients without PE.

### Interpretation of novel findings

We included patients with D-dimer values higher than 1 μg/mL, which have been associated with disease severity and in-hospital mortality in patients with COVID-19 infection. This is a relatively common condition, ranging from 40–70% in the largest retrospective studies [3,5]. PE prevalence in our patients was particularly high, since the incidence of PE in acutely ill hospitalized medical patients on prophylaxis with enoxaparin is very low (0–0.4%) [20–22]. Our data do not allow us to distinguish whether PE started before or during admission. Only one of our patients, allocated in the non-PE group, was not on enoxaparin prophylaxis during hospitalization. We also found a trend to longer time from COVID-19 infection symptoms onset to hospital admission among those patients with PE. Moreover, PE patients were older than those ones without PE, but we did not find significant differences in the number of PE risk factors between both groups. It could be speculated that up-regulation of procoagulant activity in COVID-19 infection may additively or synergistically increase the risk of PE, although this hypothesis needs to be proven in further studies. One might think, that the proportion of patients with PE could be higher at the time when blood tests (D-dimer) were performed at clinician discretion, compared to the first days when laboratory tests were performed according to protocol, resulting in a bias of selection. However, the proportion of patients with PE diagnosed during the first days was higher (66.7%) than the proportion of PE patients diagnosed during follow-up (42.9%). Furthermore, the time from onset of symptoms to CTPA was not different in the PE group vs. the non-PE group (p = 0.802).

The coexistence of pneumonia and PE has been known for years, and it is still today a diagnostic challenge [23]. Data from the international cohort RIETE showed that patients with respiratory infections had higher risk of PE than patients with other types of infections [24]. Some other studies showed that up to 90% of patients admitted to the hospital for pneumonia had high procoagulant markers, with D-dimer being one of the most common [25]. D-dimer is a very useful biomarker for excluding PE in the general population when clinical probability is low. However, it is usually not useful to diagnose the presence of PE since other inflammatory conditions may increase its value. Moreover, this biomarker is not suitable to rule out or confirm PE in patients with pneumonia in whom D-dimer levels are also increased [26]. This same circumstance seems to occur in COVID-19 disease.

The mechanisms by which COVID-19 could increase the risk of PE are unknown. PE is the result of Virchow’s classic risk triad, namely vascular endothelial impairment, stasis of blood flow, and/or increased coagulability. COVID-19 could hypothetically affect all the 3 mechanistic pathways [27]. SARS-CoV-2 appears to use the angiotensin-converting enzyme receptor 2 to enter into lung cells. These proteins are also expressed in endothelial cells, and therefore this type of cells could be a target for the virus. Furthermore, hypoxia, which is present in a significant proportion of seriously ill patients with COVID-19, may lead to thrombosis by increasing blood viscosity and by increasing systemic inflammatory response [28]. Lastly, patients with severe COVID-19 pneumonia can trigger a state of sepsis that can induce the release of inflammatory cytokines such as IL-6, IL-8, TNF-α, among others that can promote the activation of a hypercoagulability disorder [28]. Some patients even have a more prominent inflammatory response, which is associated to high D-dimer levels [2–8]. We found higher D-dimer levels, baseline D-dimer-to-ferritin, D-dimer-to-LDH and D-dimer-to-CRP ratios in patients with PE compared to those without PE. This secondary analysis could represent that despite the clear link between inflammation and subsequent activation of coagulation in severe COVID-19, patients with higher D-dimer values in relation to their inflammatory response could be at a higher risk of PE. Nonetheless, further studies with larger sample size are needed to explore this hypothesis at a deeper level. Besides, we also found a significantly higher platelet count in the group of COVID-19 patients with PE compared with non-PE subjects, although median values were within normal limits. Increase of both platelets and acute phase reactants has been detected due to the severe inflammatory state of these patients [5,8,29]. Interestingly, Yin et al. [30] found higher mortality rates, and platelet count in consecutive patients with COVID-19 severe pneumonia compared with non-COVID pneumonia patients. Nevertheless, an alternative hypothesis is that the virus induce a direct alveolar inflammation which triggers hemostasis activation causing direct vascular thrombosis in the lungs [31]. Recent data showing that consecutive COVID-19 pneumonia patients and D-dimer >1 μg/mL did not have higher frequency of DVT to that reported in general internal medicine subjects could support this latter theory [32,33].

### Previous studies

There are only few studies that have evaluated PE in patients with COVID-19 infection. A retrospective study evaluated 106 patients who underwent a CTPA during hospitalization and they confirmed PE in 32 subjects. Furthermore, they found higher D-dimer levels in PE patients compared with those patients without PE [13]. Similarly, Klok et al. [12] found a cumulative incidence of arterial or venous thrombosis of 31% in patients with COVID-19 pneumonia who had been admitted to ICU. PE was diagnosed in 25 patients and it was the most common thrombotic disorder. Unfortunately, no D-dimer values were reported. One more retrospective study detected Venous thromboembolism (VTE) in 35 of 74 (11 with PE) ICU patients despite being on thrombosis prophylaxis [16]. Interestingly, mean D-dimer values were associated with higher VTE risk. A recent analysis from a French group showed that the rate of thromboembolic complications in 150 COVID-19 patients with ARDS was much higher (11.7%) than what observed in a historical control group of non-COVID-19 ARDS patients (2.1%) despite anticoagulation [15]. Although these preliminary data suggested a high prevalence of PE in severe patients with COVID-19 pneumonia, true prevalence was not known, since diagnostic tests were only performed if thrombotic complications were clinically suspected. On contrary, we diagnosed underlying PE in 50% of patients with COVID-19 pneumonia and D-dimer levels higher than 1 μg/mL, regardless of clinical suspicion.

### Clinical implications

PE is a challenging diagnosis that may be ignored because of non-specific clinical presentation. However, early diagnosis is essential, since well-timed treatment is highly effective and significantly influences clinical outcomes [34].

Previous studies in non COVID-19 patients have shown that PAOI was correlated to PaO2, right ventricle dysfunction, and to systolic blood pressure in PE [35]. Moreover, it has been reported that patients with higher PAOI, had an increased short-term risk of death [36]. It should acknowledge that 60% of our patients had principally segmental and subsegmental vessels and its clinical relevance it is unknown, but we found significant correlations between the PAOI and D-dimer values, therefore, those patients with higher D-dimer values were found to have more clot burden in pulmonary arteries (Fig 2). As mentioned above, some retrospective studies found that a high D-dimer level was a mortality risk factor, and that anticoagulation treatment in severe COVID-19 could improve the survival rate [3,9]. Besides, a recent autopsy study included 12 consecutive patients who died of COVID-19. Interestly, DVT was detected in 7 of them, and PE was the direct cause of death in 4 patients (one third), which was not detected while they were still alive [10]. Taken together, all these preliminary available data could support the hypothesis that severe COVID-19 infection, as a procoagulant disease, may have an increased risk of PE, which could represent one of the reasons for the increased morbidity and mortality. However, to clarify the influence of D-dimer levels, PE, and PAOI as mortality risk factors in patients with COVID-19 pneumonia, more prospective and rigorous investigations are still needed.

### Strengths and limitations

The present study has several strengths. Firstly, this is the first study to demonstrate prospectively the role of PE in the pathogenesis of COVID-19 pneumonia in patients without a clear suspicion; and secondly, all the patients were exhaustively selected and characterized from the clinical, imaging and inflammatory point of view. Yet, as in any study, there are some potential limitations that deserve comment. Firstly, the sample size was small. However, the magnitude of the signal and its clinical implication precluded us to extend the observation period. Secondly, although most patients had neither signs nor symptoms of DVT, lower limb venous statuses were not routinely and objectively investigated, therefore, asymptomatic events could not be ruled out. Finally, the design of the present study does not permit to determine if PE was a direct consequence of COVID-19 infection, systemic inflammation or hypoxia. Moreover, it was not possible to assess whether PE happened previous or during hospitalization.

## Conclusions

In summary, our results demonstrate a PE prevalence of 50% in patients with COVID-19 pneumonia and D-dimer values higher than 1 μg/mL. We consider that these findings could have clinical relevance in the management of patients with COVID-19, since many of these patients would benefit from starting anticoagulant therapy, which could have a beneficial impact on the prognosis. These data should encourage the scientific community to perform further and larger studies to clarify the impact of an early PE diagnosis, and a prompt treatment initiation on the prognosis of patients with COVID-19 pneumonia.

## Supporting information

S1 Table Baseline laboratory data. Values represent median (IQR). Abbreviations: ALT, alanine aminotransferase; PT, prothrombin time; PaO2, partial pressure of arterial blood oxygen; FiO2, fractional inspired oxygen; PaCO2, partial pressure of arterial blood carbon dioxide.
(DOCX)

S2 Table Sensitivity, specificity, PPV and NPV of different D-dimer cut-off points for PE diagnosis in Covid-19 pneumonia patients. Abbreviations: PPV, Positive predictive values; NPV, negative predicted values.
(DOCX)

S1 Fig Biomarkers ratio by group of patients. Abbreviations: IQR: interquartile range; PE: pulmonary embolism; DD: D-dimer; LDH: lactate dehydrogenase; RCP: reactive C-protein. Units used: D-dimer (μg/mL), Ferritin (ng/mL), LDH (U/L), RCP (mg/dL).
(TIF)
