# Supplementary material for: Prevalence of pulmonary embolism in patients with COVID-19 pneumonia and high D-dimer values: A prospective study
Source: PLoS One. 2020 Aug 25;15(8):e0238216. doi: 10.1371/journal.pone.0238216 (PMC7447036; doi:10.1371/journal.pone.0238216)
Supplement: S1 Table — Values represent median (IQR). Abbreviations: ALT, alanine aminotransferase; PT, prothrombin time; PaO2, partial pressure of arterial blood oxygen; FiO2, fractional inspired oxygen; PaCO2, partial pressure of arterial blood carbon dioxide. (DOCX) [file pone.0238216.s001.docx]

S1 Table. Baseline laboratory data

|  | All patients  (n=30) | PE patients  (n=15) | Non-PE patients (n=15) | p value |
| --- | --- | --- | --- | --- |
| Blood count, baseline |  |  |  |  |
| Hemoglobin, g/dL | 13.2 (12.1-14.6) | 12.9 (11.7-14.2) | 14 (12.1-15.3) | 0.289 |
| Leucocytes, 10^3^/µL | 7.21 (5.3-9.73) | 7.29 (6.03-11.6) | 5.76 (4.36-9.56) | 0.165 |
| Neutrophils, 10^3^/µL | 6.09 (3.92-7.92) | 6.23 (4.63-9.16) | 4.99 (3.02-7.73) | 0.237 |
| Lymphocytes, 10^3^/µL | 0.96 (0.6-1.27) | 1.05 (0.59-1.29) | 0.95 (0.6-1.23) | 0.820 |
| Platelets, 10^3^/µL | 216.0 (171.3 – 315.5) | 298.0 (181.0 – 338.0) | 184.0 (165.0 – 256.0) | 0.034 |
| Biochemical profile, baseline | | | | |
| Glucose, mg/dL | 126 (101-163) | 132 (102-186) | 119 (92-143) | 0.305 |
| ALT, U/L | 27 (16.25-62.25) | 48 (17-64) | 26 (13.5-60) | 0.356 |
| Urea, mg/dL | 35 (27-47) | 35 (30-47) | 31 (21-47) | 0.271 |
| Creatinine, mg/dL | 0.79 (0.66-0.92) | 0.87 (0.66-1.01) | 0.77 (0.66-0.86) | 0.340 |
| Sodium, mEq/L | 137 (135-139) | 137 (135-139) | 137 (135-139) | 0.965 |
| Potassium, mEq/L | 4 (3.6-4.7) | 4.3 (3.6-4.63) | 3.9 (3.6-4.8) | 0.678 |
| Coagulation function, baseline | | | | |
| PT,s | 13.6 (12.25-14.93) | 12.7 (12.1-15) | 10.3 (12.6-14.9) | 0.787 |
| PT, % | 74.5 (65-86.8) | 82 (65-89) | 74 (65-83) | 0.771 |
| Arterial blood test, baseline | | | | |
| PaO_2_/FiO_2_ ratio | 264 (211-343) | 252 (192-321) | 293 (219-354) | 0.455 |
| pH | 7.49 (7.46-7.51) | 7.49 (7.47-7.49) | 7.49 (7.41-7.51) | 0.535 |
| PaO_2_, mmHg | 63 (51.5-75.3) | 61 (52-77.3) | 65 ( 49.3-75.8) | 0.979 |
| PaCO_2_, mmHg | 29.5 (26-31) | 28.5 (26-31) | 30 (24.5-31.8) | 0.697 |
|  | | | | |
