# Supplementary material for: Prevalence of pulmonary embolism in patients with COVID-19 pneumonia and high D-dimer values: A prospective study
Source: PLoS One. 2020 Aug 25;15(8):e0238216. doi: 10.1371/journal.pone.0238216 (PMC7447036; doi:10.1371/journal.pone.0238216)
Supplement: S2 Table — Abbreviations: PPV, Positive predictive values; NPV, negative predicted values. (DOCX) [file pone.0238216.s002.docx]

S2 Table. Sensitivity, specificity, PPV and NPV of different D-dimer cut-off points for PE diagnosis in Covid-19 pneumonia patients

|  | D dimer >1 µg/mL | D dimer >1.5 µg/mL | D dimer >2 µg/mL | D dimer >2.5 µg/mL | |
| --- | --- | --- | --- | --- | --- |
| Sensitivity | 100% | 93% | 87% | 80% | |
| Specificity | 0% | 13% | 40% | 53% | |
| PPV | 50% | 52% | 59% | 63% | |
| NPV | 0% | 67% | 75% | 73% | |
|  | | | | |  |
