# Supplementary figures and images for: Prevalence of pulmonary embolism in patients with COVID-19 pneumonia and high D-dimer values: A prospective study
Source: PLoS One. 2020 Aug 25;15(8):e0238216. doi: 10.1371/journal.pone.0238216 (PMC7447036; doi:10.1371/journal.pone.0238216)

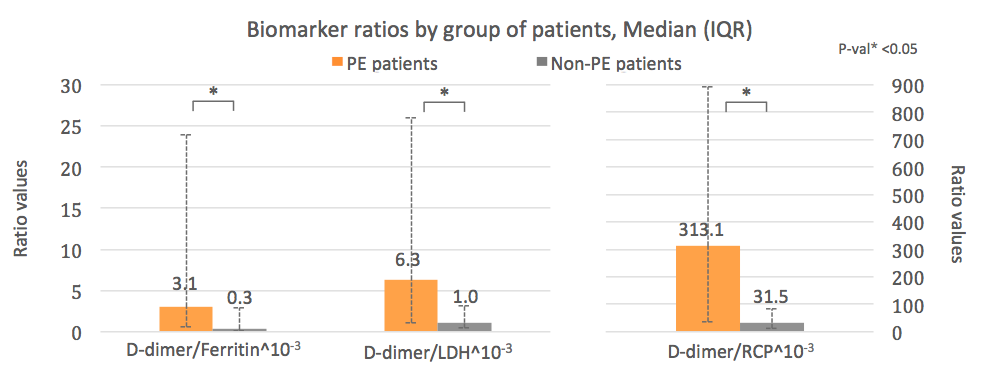

Supplement: S1 Fig — Abbreviations: IQR: interquartile range; PE: pulmonary embolism; DD: D-dimer; LDH: lactate dehydrogenase; RCP: reactive C-protein. Units used: D-dimer (μg/mL), Ferritin (ng/mL), LDH (U/L), RCP (mg/dL). (TIF) [file pone.0238216.s003.tif]
